# Impact of Finerenone in Patients with Heart Failure and Reduced/Mildly Reduced Ejection Fraction, Diabetes Mellitus, and Chronic Kidney Disease

**DOI:** 10.3390/jcm14227997

**Published:** 2025-11-11

**Authors:** Yuki Hida, Teruhiko Imamura, Koichiro Kinugawa

**Affiliations:** Second Department of Internal Medicine, University of Toyama, Toyama 930-0194, Japan

**Keywords:** heart failure, mineralocorticoid receptor antagonist, reverse remodeling

## Abstract

**Background**: Heart failure (HF) remains a major global health challenge with substantial morbidity, mortality, and healthcare burden. Finerenone, a novel non-steroidal mineralocorticoid receptor antagonist (MRA), has demonstrated cardiovascular and renal benefits in patients with diabetes mellitus (DM) and chronic kidney disease (CKD), as well as reduced HF events, in patients with HF with preserved or mildly reduced ejection fraction (HFpEF/HFmrEF). However, its efficacy and safety in patients with HF with reduced EF (HFrEF) or HFmrEF, DM, and CKD, remain unclear. Notably, finerenone is currently reimbursed only for DM and CKD but not for HF itself. **Methods**: We conducted a retrospective, single-center study of patients with HFrEF/HFmrEF who received finerenone between August 2022 and June 2025. Clinical data were obtained 6 months before, at baseline, and 6 months after the initiation of finerenone. The primary outcome was the change in serum NT pro-B-type natriuretic peptide (BNP). **Results**: Among 37 patients screened, 22 who initiated finerenone on a de novo basis were included. Median age was 75 years, 73% were male, and all had DM and CKD, which are current indications of finerenone. NT pro-BNP decreased significantly during the on-treatment period compared with the pre-treatment period (*p* < 0.001). Left ventricular end-diastolic diameter and left ventricular ejection fraction remained unchanged during the pre-treatment period but improved significantly during the on-treatment period (*p* < 0.001 for both). Renal function and serum potassium remained stable during the whole study period (*p* > 0.05 for both). **Conclusions**: In this real-world study of patients with HFrEF/HFmrEF complicated by DM and CKD, finerenone was associated with significant improvements in NT pro-BNP and cardiac remodeling indices without worsening renal function or hyperkalemia.

## 1. Background

Heart failure (HF) remains a major global health challenge, imposing substantial morbidity, mortality, and healthcare burden [1,2]. In patients with HF with reduced ejection fraction (HFrEF), guideline-directed medical therapy is anchored by a foundational quadruple regimen; renin-angiotensin system inhibitor/angiotensin receptor-Neprilysin inhibitor, sodium–glucose cotransporter 2 inhibitor, and mineralocorticoid receptor antagonist (MRA) [1,3,4]. Among these pharmacological pillars, MRA represents a key component targeting maladaptive neurohormonal activation and myocardial fibrosis. Landmark trials such as RALES and EMPHASIS-HF have consistently demonstrated its survival benefits and reduction in HF hospitalizations [5,6,7].

Despite their proven efficacy, the real-world use of conventional steroidal MRA, such as spironolactone and eplerenone, is frequently constrained by the risks of hyperkalemia and renal dysfunction [8,9]. This underutilization is particularly pronounced in patients with concomitant diabetes mellitus (DM) and chronic kidney disease (CKD), populations at heightened risk of both cardiovascular and renal events, particularly under the use of MRA [10,11]. Consequently, there remains a substantial therapeutic gap between the guideline recommendations and real-world clinical practice in these high-risk patients.

Finerenone, a novel non-steroidal selective MRA, has recently emerged as a promising alternative. The FIDELIO-DKD and FIGARO-DKD trials established its ability to improve cardiovascular and renal outcomes in patients with type 2 DM and CKD [12,13]. More recently, the FINEARTS-HF trial demonstrated that finerenone reduces the risk of HF events in patients with HF with preserved or mildly reduced ejection fraction (HFpEF/HFmrEF) [14]. In addition, finerenone has been reported to exert a diuretic-sparing effect, potentially mitigating the adverse prognostic impact of escalating loop diuretic use [15]. Its favorable safety profile and lower incidence of hyperkalemia compared with traditional MRAs further enhance its clinical attractiveness, particularly for patients with renal impairment.

Nevertheless, the efficacy and safety of finerenone in patients with HFrEF or HFmrEF complicated by DM and CKD remain uncertain, as these patients are often excluded from clinical trials and face unique therapeutic challenges. For instance, a pooled analysis of FIDELIO-DKD, FIGARO-DKD, and FINEARTS-HF trials identified only 237 patients who had HFmrEF/HFpEF, DM, and CKD among a total of 18,991 enrolled participants, and importantly, none of these patients had HFrEF [16]. In that analysis, finerenone numerically favored a reduction in cardiovascular death versus placebo.

Furthermore, finerenone is currently not reimbursed for HF itself in Japan. Finerenone has an indication only in patients with DM and CKD, although some of them might have incidentally comorbid HF. Given the overlapping pathophysiology of HF, DM, and CKD—collectively termed the “cardio-renal-metabolic syndrome”—a comprehensive understanding of finerenone’s role across this spectrum is urgently needed.

Clarifying the role of finerenone in this high-risk population is therefore of major clinical importance. Accordingly, the present study aimed to retrospectively evaluate the real-world efficacy, safety, and tolerability of finerenone in patients with HFrEF or HFmrEF and coexisting DM and CKD.

## 2. Methods

### 2.1. Patient Selection

Patients with HF who received finerenone for DM and CKD between August 2022 and June 2025 were eligible for the present retrospective study. Notably, finerenone had an indication and was reimbursed only in patients with DM and CKD in Japan at the time when the present study was conducted. Thus, the eligible patients had HF incidentally and received finerenone for their DM and CKD.

Patients with hyperkalemia, those dependent on hemodialysis, and those with symptomatic hypotension did not receive finerenone and were not included. Patients who had received other types of MRAs and were converted to finerenone were excluded (i.e., only the patients who received finerenone on a de novo basis were eligible). Patients with left ventricular ejection fraction (LVEF) >50% were excluded. Patients with critical data lacking were also not included.

The diagnosis of HF was made according to the current guidelines of Japanese Circulation Society [17]. The study protocol was approved by the local institutional review board. Written informed consent was waived given the retrospective nature of this study and the opt-out method.

### 2.2. Study Design

Clinical data were retrieved at three time points: six months before the initiation of finerenone, at the time of finerenone initiation, and six months after the initiation of finerenone. The period before the initiation of finerenone was defined as a pre-treatment period, whereas the period following the initiation of finerenone was defined as an on-treatment period. Importantly, the pre-treatment period was assumed as a control.

The primary outcome was the improvement of serum NT pro-B-type natriuretic peptide (BNP) levels. The secondary outcomes were echocardiographic parameters, including left ventricular end-diastolic diameter and LVEF, and laboratory data, including estimated glomerular filtration rate and serum potassium level. The trajectory of these parameters was compared between the pre-treatment (control) and on-treatment periods.

### 2.3. Clinical Management

Finerenone was initiated for DM and CKD, which was the only reimbursed indication in Japan at the time when the present study was conducted. Patients with hyperkalemia and those with end-stage renal function or dependent on hemodialysis did not receive finerenone.

Finerenone was initiated at 20 mg/day when patients had an estimated glomerular filtration rate ≥60 mL/min/1.73 m^2^. The initial dose was decreased to 10 mg/day when patients had <60 mL/min/1.73 m^2^ of estimated glomerular filtration rate. The dose was considered to be up-titrated to 20 mg/day after 4 weeks of careful observation. The adjustment of finerenone dose was at the discretion of the attending physicians, who considered patients’ blood pressure, serum potassium, and estimated glomerular filtration rate levels. The adjustment of doses of other HF medications was also at the discretion of the attending physicians.

### 2.4. Data Collection

Demographics, laboratory data, echocardiographic, and medication data at the time of finerenone initiation (defined as baseline) were retrieved. Similar clinical data, except for demographic data, were retrieved six months before and after the initiation of finerenone (pre-treatment period and on-treatment period). For the measurement of echocardiographic parameters, the board-certified cardiologist independently adjudicated the results. LVEF was calculated by using the modified Simpson method and reported as an exact number.

### 2.5. Statistical Analyses

Statistical analyses were performed using SPSS Statistics 25 (SPSS Inc., Armonk, IL, USA). Two-sided *p*-values < 0.05 were considered statistically significant. Continuous variables were stated as median (25% interquartile, 75% interquartile) given the small sample size. Categorical variables were stated as numbers and percentages. Trends were assessed by using the Friedman test and post hoc Wilcoxon signed-rank test, or Cochran Q test and post hoc McNemar test. Logistic regression analyses were performed to investigate factors associated with clinical improvement, which was defined as any decrease in serum NT pro-BNP level and >5% increase in LVEF at 6-month follow-up. Variables with *p* < 0.10 in the univariable analysis were included in the multivariable analysis with a forced method.

## 3. Results

### 3.1. Baseline Characteristics

A total of 37 patients with HFrEF or HFmrEF received finerenone between August 2022 and June 2025. Of them, 13 patients who had received other types of MRA and switched them to finerenone and 2 with critical data deficiencies were excluded. Finally, 22 patients were included in the present retrospective study.

Patients’ baseline characteristics at the time of finerenone initiation are summarized in Table 1. Median age was 75 (72, 80) years and 16 (73%) were males. All patients had DM and CKD, which was the only reimbursed indication of finerenone. Furthermore, all patients had HFrEF or HFmrEF (LVEF < 50%). The etiologies of HF were as follows: 15 ischemic cardiomyopathy, 3 dilated cardiomyopathy, 3 hypertensive heart disease, and 1 valvular disease. Estimated glomerular filtration rate on median was 42.3 (28.9, 54.2) mL/min/1.73 m^2^. The common logarithm of serum NT pro-BNP on median was 3.33 (2.77, 3.41) pg/mL. Median LVEF was 41 (36, 48)%.

### 3.2. Feasibility of Finerenone Therapy

All patients completed 6-month finerenone therapy, except for 2, who terminated finerenone at day 32 and day 150, respectively, due to advanced renal impairment (estimated glomerular filtration rate from 27.2 to 10.0 mL/min/1.73 m^2^ and from 28.9 to 19.5 mL/min/1.73 m^2^, respectively).

### 3.3. The Trajectory of Serum NT Pro-BNP Level

**Figure 1:**
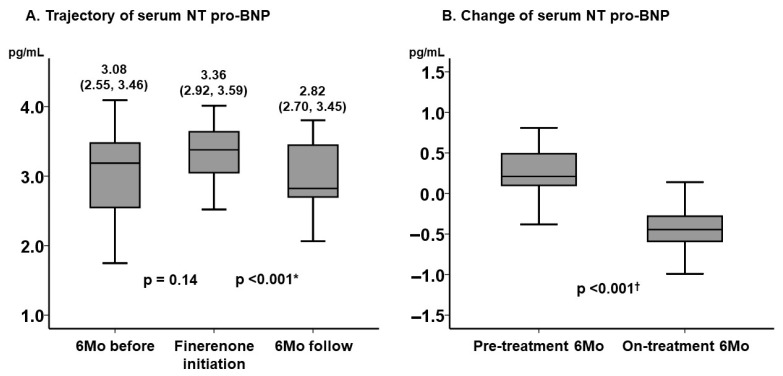
Trajectory of serum NT pro-BNP level (**A**) and the change in serum NT pro-BNP level before and after the initiation of finerenone (**B**). Serum NT pro-BNP was expressed as a form of common logarithm. The level of NT pro-BNP remained unchanged during 6-month period before the initiation of finerenone and decreased significantly during 6-month finerenone treatment period. The degree of improvement in NT pro-BNP was significantly higher during the on-treatment period. * *p* < 0.05 by post hoc Wilcoxon signed-rank test following the Friedman test; ^†^ *p* < 0.05 by Mann–Whitney U test. BNP, B-type natriuretic peptide.

Serum NT pro-BNP level remained unchanged during the pre-treatment period (*p* = 0.14), whereas it decreased significantly during the on-treatment period (*p* < 0.001) (Figure 1A). The degree of improvement in NT pro-BNP level was significantly greater during the on-treatment period than the pre-treatment period (*p* < 0.001) (Figure 1B).

### 3.4. The Trajectory of Key Clinical Parameters

**Figure 2:**
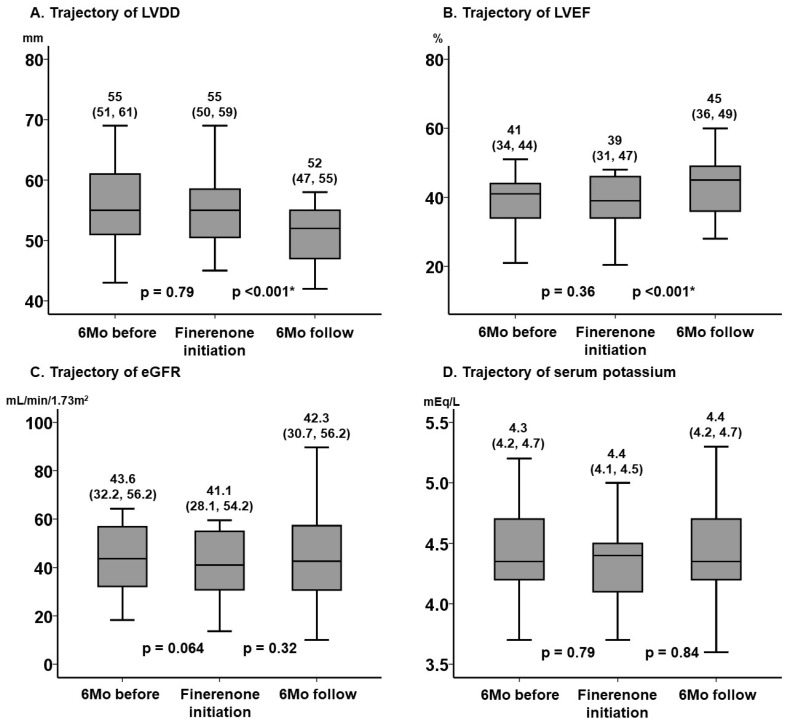
Trajectory of LVDD (**A**), LVEF (**B**), eGFR (**C**), and serum potassium (**D**) before and after the initiation of finerenone. These key clinical parameters remained unchanged during the pre-treatment 6-month observation period without finerenone. During the 6-month on-treatment period, LVDD and LVEF improved significantly, whereas eGFR and serum potassium level did not worsen significantly. * *p* < 0.05 by post hoc Wilcoxon signed-rank test following the Friedman test. LVDD, left ventricular end-diastolic diameter; LVEF, left ventricular ejection fraction; eGFR, estimated glomerular filtration rate.

The key echocardiographic parameters, including left ventricular end-diastolic diameter and LVEF, remained unchanged during the 6-month pre-treatment period (*p* > 0.05 for both), whereas they improved significantly during the 6-month on-treatment period (*p* < 0.001 for both) (Figure 2A,B).

The key laboratory parameters, including estimated glomerular filtration rate and serum potassium, remained unchanged during the 6-month pre-treatment period (*p* > 0.05 for both). The worsening of renal function and the increase in serum potassium were not significant during the 6-month on-treatment period (*p* > 0.05 for both) (Figure 2C,D).

### 3.5. Factors Associated with Clinical Improvement

Prognostic impact of the potential baseline characteristics on the clinical improvement, which was defined as any decrease in serum NT pro-BNP levels and an increase in LVEF >5% at 6-month follow-up, was assessed. Younger age (odds ratio 0.77, 95% confidence interval 0.62–0.96, *p* = 0.019) and lower estimated glomerular filtration rate (odds ratio 0.84, 95% confidence interval 0.71–0.99, *p* = 0.042) were independently associated with the endpoint achievement (Table 2).

### 3.6. The Trajectory of Other Clinical Parameters

The trajectory of other clinical parameters is summarized in Table 3. All parameters remained statistically unchanged both during the pre-treatment and on-treatment period (*p* > 0.05 for all). Notably, the administration rates of HF medications remained statistically unchanged during the whole observation period, although the actual numbers were numerically fluctuated (*p* > 0.05 for all).

## 4. Discussion

In this retrospective, single-center study of patients with HFrEF or HFmrEF complicated by DM and CKD, initiation of finerenone was associated with significant improvements in NT pro-BNP levels and indices of left ventricular remodeling, including reduced left ventricular end-diastolic diameter and increased LVEF, without deterioration in renal function or development of hyperkalemia. Importantly, we used the pre-treatment period as a self-control.

These findings suggest that finerenone may provide meaningful cardiovascular benefit in this high-risk population (i.e., the combination of HFrEF/HFmrEF, DM, and CKD). Importantly, the favorable changes observed during the 6-month on-treatment period contrasted with the stability of biomarkers and echocardiographic parameters during the pre-treatment period, reinforcing the likelihood that finerenone was responsible for the beneficial trajectory rather than reflecting a background trend. This self-controlled design strengthens the internal validity of our findings and suggests that even in the absence of randomization, temporal consistency supports a treatment effect.

Our results are consistent with the well-established efficacy of conventional steroidal MRA in HFrEF. The RALES trial demonstrated a 30% reduction in all-cause mortality with spironolactone in patients with advanced systolic HF, while EMPHASIS-HF confirmed the prognostic value of eplerenone in mild HFrEF, and EPHESUS showed improved outcomes in patients with left ventricular dysfunction after myocardial infarction [5,6,18]. Together, these landmark studies firmly established MRA as a cornerstone of therapy in HFrEF. However, translating these benefits into routine practice has remained difficult, particularly among patients with renal dysfunction or diabetes, where the risk-benefit balance is often unfavorable, particularly in elderly patients with multiple comorbidities. Our data suggest that finerenone could narrow this evidence–practice gap by offering a safer MRA profile suitable for fragile patients.

The real-world adoption of conventional MRA remains suboptimal, primarily because of concerns regarding hyperkalemia and renal dysfunction [8,9]. This therapeutic gap is particularly relevant in patients with concomitant DM and CKD, populations at intrinsically higher risk of both cardiovascular and renal events. In this regard, our observation that renal function and serum potassium level remained stable during finerenone therapy, despite a median baseline eGFR of only 42.3 mL/min/1.73 m^2^, should be clinically meaningful. The absence of a significant rise in serum potassium level highlights the potential of finerenone to overcome major barriers to broader MRA use in such high-risk patients. This finding aligns with prior pharmacovigilance data and provides real-world reassurance that finerenone may permit continued neurohormonal blockade even in patients traditionally excluded from MRA therapy. The finding is particularly advantageous in patients with HFrEF, who concomitantly receive other renin–angiotensin–aldosterone system inhibitors that increase serum potassium levels.

Pharmacologically, finerenone differs from spironolactone and eplerenone in its greater receptor selectivity, reduced lipophilicity, and lower tissue accumulation, features that may contribute to a more favorable safety profile [19]. Beyond these pharmacokinetic advantages, preclinical studies suggest that finerenone exerts potent anti-fibrotic and anti-inflammatory effects, which may translate into benefits for cardiac remodeling [19]. The FIDELIO-DKD and FIGARO-DKD trials demonstrated that finerenone reduced cardiovascular death and kidney disease progression in patients with DM and CKD, establishing its dual cardiorenal protective effects [12,13]. More recently, the FINEARTS-HF trial extended this evidence to patients with HFmrEF/HFpEF, showing reductions in the composite of HF events and cardiovascular death [14]. A pre-specified analysis further revealed that finerenone reduced the need for loop diuretic intensification, suggesting a diuretic-sparing effect [15].

These observations are of particular clinical importance, as escalating loop diuretic doses are strongly associated with poor outcomes and progressive renal impairment [20,21]. Our finding that cardiac function improved without renal deterioration lends support to the hypothesis that finerenone may interrupt the vicious cycle of congestion, kidney dysfunction, and escalating diuretic requirements. Mechanistically, finerenone’s anti-inflammatory and anti-fibrotic actions may contribute to improved myocardial compliance, reduced ventricular stiffness, and better natriuretic efficiency, thereby enhancing both cardiac and renal homeostasis.

A notable finding in our study was that younger age and lower baseline eGFR were independently associated with clinical improvement, defined as reductions in NT pro-BNP and significant increases in LVEF. This paradoxical result suggests that patients with more advanced renal dysfunction may derive greater benefit from finerenone, further highlighting its potential value in populations traditionally considered unsuitable for treatment with MRA. The younger age may be associated with the potential of organ reversibility. Younger patients generally have greater organ reversibility than older ones.

Although speculative, one possible explanation is that non-steroidal MRA, such as finerenone, might exert reno-protective effects that counterbalance the risks of hyperkalemia and worsening kidney function, thereby allowing continued neurohormonal blockade in advanced CKD. Alternatively, it is conceivable that patients with greater neurohormonal activation or fibrotic burden—features common in advanced CKD—may be more responsive to the pleiotropic effects of finerenone. Further mechanistic studies exploring the interplay between renal dysfunction, inflammation, and MRA responsiveness are warranted.

The clinical challenge of managing patients with HFrEF/HFmrEF complicated by DM and CKD cannot be overstated. These individuals not only carry a higher risk of adverse outcomes but are also less likely to receive comprehensive guideline-directed medical therapy, reflecting the tension between efficacy and safety in clinical decision-making. Underutilization of MRAs remains a persistent problem, often driven by fear of hyperkalemia and worsening renal function [8,9]. Our results suggest that finerenone could represent a viable alternative in such patients, enabling the survival benefits of treatment therapy with MRA to be extended more broadly. Moreover, the stability of concomitant therapies, including beta-blocker, angiotensin receptor neprilysin inhibitor, and sodium–glucose cotransporter 2 inhibitor, during the study period, suggests that the improvements observed are attributable to finerenone itself rather than background therapy changes. This is particularly relevant in the era of comprehensive quadruple therapy, where maintaining multi-drug adherence without compromising renal safety is an emerging priority in heart failure management.

Our study also raises the possibility that finerenone could serve as an effective MRA option across the LVEF spectrum. While the benefits of spironolactone and eplerenone are firmly established in HFrEF, evidence in HFpEF has been inconsistent. The TOPCAT trial reported a reduction in HF hospitalizations with spironolactone but failed to improve its primary composite outcome, in part due to regional variability and adherence concerns [22]. In contrast, FINEARTS-HF provided robust evidence for finerenone in HFmrEF and HFpEF, leading to recent guideline endorsements [14]. Our data, albeit exploratory, complement these findings by suggesting that finerenone may also be effective in patients across the reduced LVEF spectrum, including those with HFrEF. If validated in larger prospective cohorts, finerenone would stand out as a unique therapy with the demonstrated benefits spanning the continuum of LVEF phenotypes.

Taken together, our findings provide real-world evidence supporting the use of finerenone in high-risk patients with HFrEF or HFmrEF complicated by DM and CKD. By improving biomarkers and cardiac structure without compromising renal function or serum potassium balance, finerenone may help bridge a major gap in the application of guideline-directed medical therapy. These results align with prior evidence in HFmrEF/HFpEF and extend the potential utility of finerenone to patients with reduced LVEF. Although our study is limited by its retrospective design, small sample size, lack of a control group, and short follow-up, the consistent improvements in surrogate markers underscore the need for further research. Prospective randomized controlled trials focusing on this high-risk population are warranted to confirm the efficacy and safety of finerenone and to determine whether surrogate improvements translate into reductions in mortality and hospitalization.

### Limitations

Several limitations of our study should be acknowledged. First, the retrospective design and small sample size substantially limit the strength and generalizability of our conclusions, raising the possibility of residual confounding, despite the use of the pre-treatment period as a historical control. Notably, particularly given the small sample size, the statistical non-significance in the inter-group comparison does not guarantee the similarity. We assumed all continuous variables as categorical variables, irrespective of their distribution normality, given the small sample size. We emphasize that the administration of finerenone in patients with HF itself has not been reimbursed in Japan so far. We included the patients who had HFrEF/HFmrEF by chance and received finerenone for DM and CKD. This is a major reason for the small sample size in the present study. Accordingly, patient selection was not based on a standardized protocol, and prescribing bias may have influenced the observed outcomes. Moreover, because data collection relied on clinical records, unmeasured confounders such as medication adherence, dietary sodium intake, or temporal variations in hemodynamic status may have affected the results. Conversely, we are proud of the present study as a proof-of-concept that was conducted prior to the reimbursement of finerenone for HF Further larger-scale studies are warranted to validate our findings, probably following the reimbursement of finerenone for HF.

Second, the absence of a prospective randomized control group precludes definitive attribution of the observed benefits solely to finerenone. Although the comparison with a pre-treatment period partially addresses this concern, the inherent risk of time-dependent effects or regression to the mean cannot be entirely excluded. Furthermore, because background therapy was optimized according to contemporary standards, the incremental contribution of finerenone to the observed improvements cannot be precisely quantified. A randomized design with a matched control arm would be necessary to confirm causality. Conversely, it may be ethically challenging to randomize patients with HF, DM, and CKD into the control arm (i.e., without MRAs) in the contemporary era.

Third, the follow-up duration was relatively short, and the surrogate markers such as NT pro-BNP and LVEF were assessed, rather than hard clinical endpoints such as all-cause mortality or HF hospitalization. Longer follow-up is essential to determine whether the observed biomarker and echocardiographic improvements translate into tangible clinical benefits. Additionally, the lack of repeated measurements of renal biomarkers, such as urinary albumin-to-creatinine ratio, limits our ability to evaluate the full spectrum of finerenone’s cardio-renal protective effects. LVEF should have been ideally measured by cardiac magnetic resonance imaging for accurate analyses. Patients’ quality of life and exercise capacity also remain the next concern that should be evaluated as benefits of finerenone.

Fourth, patients receiving chronic dialysis or with end-stage renal disease were not included in this study, as finerenone has not yet been recommended for this population according to the contemporary evidence. Future investigations should specifically evaluate the efficacy and feasibility of finerenone in this cohort.

Finally, although concomitant guideline-directed medical therapy, including other core HF medications, was unchanged during the study period, the potential long-term, synergistic, or adverse effects of concomitant therapies cannot be fully excluded. Drug–drug interactions, particularly with renin–angiotensin system inhibitors and SGLT2 inhibitors, may influence potassium homeostasis and renal function, necessitating careful monitoring in future investigations.

These limitations underscore the exploratory nature of our findings and the need for larger, prospective randomized trials to validate the role of finerenone in this high-risk population. Future multicenter studies with longer follow-up and more diverse patient characteristics are warranted to confirm these preliminary observations and to establish optimal patient selection criteria and monitoring strategies for finerenone therapy in clinical practice.

## 5. Conclusions

In this retrospective study of patients with HFrEF or HFmrEF with concomitant DM and CKD, the initiation of finerenone was associated with significant improvements in NT pro-BNP and indices of left ventricular remodeling, without significant worsening of renal function or development of hyperkalemia. These findings complement the existing evidence base for finerenone in HFmrEF/HFpEF and DM/CKD populations, and provide exploratory data suggesting a potential role in HFrEF.

By mitigating the risks of renal dysfunction and hyperkalemia while maintaining cardio-renal stability, finerenone may enable broader and safer application of MRA therapy in real-world HF management. Our observations also raise the possibility that finerenone’s distinct anti-fibrotic and anti-inflammatory properties could contribute to improved myocardial remodeling and more durable disease modification in high-risk patients.

## Figures and Tables

**Table 1:** Baseline characteristics at the time of finerenone initiation.

Demographics	
Age, years	75 (72, 80)
Male	16 (73%)
Body mass index	22.1 (21.1, 25.3)
History of heart failure hospitalization	16 (73%)
Comorbidity	
Diabetes mellitus	22 (100%)
Chronic kidney disease	22 (100%)
Hypertension	16 (73%)
Dyslipidemia	15 (68%)
Atrial fibrillation	6 (27%)
Laboratory data	
Hemoglobin, g/dL	14.2 (11.3, 15.8)
Serum albumin, g/dL	3.7 (3.5, 4.3)
Serum total bilirubin, mg/dL	0.6 (0.5, 0.7)
Serum potassium, mEq/L	4.4 (4.2, 4.5)
eGFR, mL/min/1.73 m^2^	42.3 (28.9, 54.2)
Serum NT pro-BNP (common logarithm), pg/mL	3.33 (2.77, 3.41)
Echocardiography data	
Left atrial volume index, mL/m^2^	37 (29, 47)
Left ventricular end-diastolic diameter, mm	57 (51, 60)
Left ventricular ejection fraction, %	41 (36, 48)
Left ventricular ejection fraction < 40%	12 (55%)
Mild or greater mitral regurgitation	1 (5%)
Mild or greater tricuspid regurgitation	0 (0%)
Heart failure medications	
Beta-blockers	18 (82%)
ACE inhibitors or ARB	6 (27%)
Angiotensin receptor neprilysin inhibitors	13 (59%)
Mineralocorticoid receptor antagonists	0 (0%)
Sodium-glucose cotransporter 2 inhibitors	19 (86%)
Loop diuretics	12 (55%)
Statins	16 (73%)
Ezetimibes	5 (23%)
Direct oral anti-coagulants	7 (32%)

Continuous variables were expressed as median (25% interquartile, 75% interquartile). Categorical variables were expressed as numbers (percentages). All patients had left ventricular ejection fraction <50%, diabetes mellitus, and chronic kidney disease. BNP, B-type natriuretic peptide; ACE, angiotensin converting enzyme; ARB, angiotensin II receptor blocker.

**Table 2:** Factors associated with clinical improvement.

	Univariable Analysis		Multivariable Analysis	
	Odds Ratio (95% CI)	*p*-Value	Odds Ratio (95% CI)	*p*-Value
Age, years	0.87 (0.75–1.01)	0.055	0.77 (0.62–0.96)	0.019 ^†^
Body mass index	1.11 (0.83–1.47)	0.49		
Non-ischemic etiology	3.67 (0.56–24.1)	0.18		
History of heart failure hospitalization	0.46 (0.07–3.09)	0.42		
Atrial fibrillation	0.83 (0.12–6.01)	0.86		
Hemoglobin, g/dL	1.01 (0.69–1.46)	0.98		
eGFR, mL/min/1.7 3m^2^	0.94 (0.87–1.01)	0.078	0.84 (0.71–0.99)	0.042 ^†^
Serum NT pro-BNP (common logarithm), pg/mL	0.71 (0.11–4.69)	0.72		
Left ventricular end-diastolic diameter, mm	1.02 (0.88–1.17)	0.83		
Left ventricular ejection fraction, %	0.94 (0.84–1.04)	0.21		
Administration of heart failure triple therapy *	0.67 (0.11–4.21)	0.67		

Variables with *p* < 0.10 in the univariable logistic regression analyses were included in the multivariable logistic regression analysis. * Consisting of beta-blockers, renin-angiotensin system inhibitors, and sodium-glucose cotransporter 2 inhibitors. ^†^ *p* < 0.05. CI, confidence interval; eGFR, estimated glomerular filtration rate; BNP, B-type natriuretic peptide.

**Table 3:** Trajectory of clinical parameters before and after the initiation of finerenone.

	Six Months Before	Finerenone Initiation	Six Months Follow	*p*-Value
Laboratory data				
Hemoglobin, g/dL	14.3 (11.0, 15.8)	14.2 (11.3, 15.8)	13.3 (11.5, 15.6)	0.56
Serum albumin, g/dL	4.1 (3.4, 4.5)	3.7 (3.5, 4.3)	4.3 (3.9, 4.5)	0.26
Serum total bilirubin, mg/dL	0.4 (0.4, 0.7)	0.6 (0.5, 0.7)	0.5 (0.4, 0.6)	0.76
Echocardiography data				
Left atrial volume index, mL/m^2^	35 (25, 44)	37 (29, 47)	36 (26, 50)	0.37
Mild or greater mitral regurgitation	0 (0%)	1 (5%)	0 (0%)	0.14
Mild or greater tricuspid regurgitation	0 (0%)	0 (0%)	0 (0%)	1.0
Medications				
Beta-blockers	16 (73%)	18 (82%)	20 (91%)	0.087
ACE inhibitors or ARB	6 (27%)	6 (27%)	6 (27%)	1.0
Angiotensin receptor neprilysin inhibitors	11 (50%)	13 (59%)	15 (68%)	0.14
Mineralocorticoid receptor antagonists *	0 (0%)	0 (0%)	0 (0%)	1.0
Sodium-glucose cotransporter 2 inhibitors	16 (73%)	19 (86%)	19 (86%)	0.17
Loop diuretics	13 (59%)	12 (55%)	13 (59%)	0.37
Statins	15 (68%)	16 (73%)	16 (73%)	0.37
Ezetimibes	4 (18%)	5 (23%)	6 (27%)	0.22
Direct oral anti-coagulants	7 (32%)	7 (32%)	6 (27%)	0.37

Clinical data at three timings were displayed: six months before the initiation of finerenone, at the time of finerenone initiation, and six months after the initiation of finerenone. * Administration of mineralocorticoid receptor antagonists, other than finerenone, was stated. No patients received mineralocorticoid receptor antagonists, except for finerenone, during the study period. Continuous variables were expressed as median (25% interquartile, 75% interquartile). Categorical variables were expressed as numbers (percentages). The trend of continuous variables was assessed by the Friedman test. The trend of categorical variables was assessed by Cochran Q test. ACE, angiotensin converting enzyme; ARB, angiotensin II receptor blocker.

## Data Availability

Data are available from the corresponding authors upon reasonable requests.
